# The causes of COVID-19: an uncontrolled experiment in evolution, causation, and public health

**DOI:** 10.1590/0074-02760250285

**Published:** 2026-10-26

**Authors:** Ricardo Waizbort

**Affiliations:** Fundação Oswaldo Cruz-Fiocruz, Instituto Oswaldo Cruz, Laboratório de Educação em Saúde e Ambiente, Rio de Janeiro, RJ

**Keywords:** COVID-19, SARS-COV-2, natural selection, artificial selection, social health

## Abstract

**BACKGROUND:**

The coronavirus disease 2019 (COVID-19) pandemic has intensified debates on causal explanation in biology, particularly regarding the integration of proximate mechanisms with broader ecological, historical, and socio-environmental determinants. Many accounts remain confined to a single explanatory level, limiting their capacity to address the complexity of pandemic emergence and control.

**OBJECTIVES:**

This paper develops a causal account of the COVID-19 pandemic by placing into dialogue *Leyes sin causa y causas sin ley en la explicación biológica*, by Gustavo Caponi, and Donald Worster’s essay ‘Another Silent Spring’.

**METHODS:**

The analysis adopts a conceptual and comparative approach, contrasting Caponi’s experimentalist account of causation, centred on intervention and invariance, with Worster’s environmental-historical interpretation of pandemic origins. The discussion is further informed by evolutionary medicine and epidemiology.

**FINDINGS:**

Caponi’s framework clarifies the epistemic role of intervention, while Worster situates the pandemic within a long-term process of ecological transformation driven by human attempts to control natural systems. Together, these perspectives reveal a tension between control as explanation and control as a source of ecological instability.

**MAIN CONCLUSIONS:**

The COVID-19 pandemic may be interpreted as a paradox of control: human efforts to manipulate biological and ecological processes generate conditions that increase systemic risk. The pandemic thus underscores the need to integrate biological, ecological, and socio-historical dimensions of causation in both explanation and prevention.

“Medicine is a social science, and politics is nothing more than medicine on a large scale.”^1^

For many scientists and physicians working in human health, understanding biological phenomena requires integrating both proximate and remote causes.^2^ ^3^ ^4^ ^5^ In the case of coronavirus disease 2019 (COVID-19), the fundamental proximate cause is the severe acute respiratory syndrome coronavirus 2 (SARS-CoV-2) itself. Immediate biological factors, such as the structure of its viral envelope proteins and their affinity for human cellular receptors, also belong to this category. Additional proximate factors, including ecological and societal variables, further contribute to transmission dynamics. By contrast, remote or distal causes are broader, historical, and more difficult to delimit. These include long-term evolutionary processes, environmental changes, and anthropogenic factors that shape the emergence of new species, adaptive traits, and zoonotic pathogens such as SARS-CoV-2.

Genetic analyses of SARS-CoV-2 samples collected from infected individuals have revealed a close phylogenetic relationship between this coronavirus and others found in bats of the *Rhinolophus* genus.^5^ ^6^Two main epidemiological hypotheses have been proposed to account for the emergence of SARS-CoV-2 in human populations: a zoonotic spillover route involving wildlife reservoirs and possible intermediate hosts, and a laboratory-associated route involving accidental exposure or release. The precise transmission route remains unresolved.^7^ ^8^ ^9^ Even so, a broad epidemiological consensus converges on a zoonotic trajectory involving bat-associated sarbecoviruses, potentially passing through intermediate animal hosts and in a process facilitated by ecological interfaces generated through wildlife trade and environmental transformation. This account draws on converging lines of evidence, including viral phylogenetics, comparative genomics, ecological studies of bat reservoirs, and epidemiological investigations of early outbreak clusters.^8^ ^9^

A remote cause of the COVID-19 pandemic thus lies in the ecological and evolutionary conditions that led to the emergence of SARS-CoV-2 and related lineages in certain bat species over tens of millions of years. Notably, the immune systems of these flying mammals differ in key ways from those of humans and other non-flying mammals.^10^ ^11^ In his 2020 article ‘Evolutionary medical insights into the SARS-CoV-2 pandemic’, published in the scientific journal *Evolution, Medicine, and Public Health*, Bernard Crespi proposes that the pandemic may be explained, in part, by a mismatch between human immune responses and viral infection. Mismatch is a foundational concept in Evolutionary Medicine, which Crespi explicitly adopts as the framework for his analysis. According to this approach, the COVID-19 pandemic stems from three interacting factors: (i) the bat immune system is characterised by low levels of inflammation and a highly efficient interferon-mediated antiviral response. Interferons are signalling proteins produced by immune cells upon detection of pathogens such as viruses, bacteria, or tumours. Their primary role is to signal nearby cells and initiate antiviral defences, including immune cell activation and the inhibition of viral replication. Unlike other mammals, bats rely minimally on inflammation as a first-line defence; (ii) “viral tactics”, mechanisms that disrupt the human interferon response, thereby enabling extensive asymptomatic and pre-symptomatic transmission, a defining feature of SARS-CoV-2’s pandemic potential; and (iii) an evolutionary trade-off between healing and harm, in which the costs of human immune responses exceed their benefits. In severe cases, this imbalance manifests as a “cytokine storm”, an extreme immune reaction involving the uncontrolled release of inflammatory cytokines capable of damaging the body’s own tissues.^10^ Evolutionary mismatches among viral adaptations, bat immune systems, and human defences should be understood as one component of a broader constellation of remote causes shaping the emergence, transmission, and pathogenicity of the virus.

Explaining a pandemic requires integrating processes that operate across different levels of organisation, from molecular interactions between viruses and host cells to large-scale ecological transformations and historical changes in human relations with the biosphere. To articulate this interpretation, I place into dialogue two seemingly distant intellectual traditions: the experimentalist conception of causality developed by Gustavo Caponi and the environmental-historical analysis of pandemic origins proposed by Donald Worster. While Caponi offers epistemological tools for understanding causal explanation in biological systems without reliance on universal laws, Worster situates the emergence of pandemics within the historical expansion of human efforts to transform and control natural systems, including the widespread manipulation of plants and animals through artificial selection and the ecological disruptions that intensify human contact with wildlife reservoirs of pathogens, such as bat-borne viruses with zoonotic potential.

Bringing these perspectives together allows us to frame the pandemic simultaneously as a biological event, an ecological phenomenon, and a historical outcome of human interventions in natural processes. From this standpoint, the COVID-19 crisis reveals a paradox of control: the same capacity that enables human societies increasingly to regulate and redirect the flows of energy across the planet, through the large-scale manipulation of ecosystems and natural resources, also generates ecological transformations that reshape the very conditions of life on Earth, including the emergence of epidemiological environments in which pandemics become more likely.

Understanding these causal relationships is not merely an explanatory task. It also has a preventive dimension. If causal explanation in biology is inseparable from the possibility of intervention, as Caponi argues, then identifying the ecological and evolutionary drivers of pandemic emergence becomes essential for designing strategies capable of reducing the likelihood of future global outbreaks. This article engages with the epistemic tensions between distinct causal frameworks, both within biology and at the boundary between biological and socio-environmental explanations.

## MATERIALS AND METHODS

*Causality, control, and crisis* - *Leyes sin causa y causas sin ley en la explicación biológica* ^12^ by the Argentine philosopher of science Gustavo Caponi was published in 2014 and thus contains no direct reference to the pandemic. Nonetheless, it offers a compelling epistemological framework grounded in an experimentalist or manipulationist conception of causality, one that proves useful for interpreting the health crisis that began in late 2019. ‘Another Silent Spring’^13^ is a brief yet powerful essay by the American environmental historian Donald Worster, published in April 2020 during the early phase of global lockdowns. In it, Worster reflects on the remote and ultimate causes of the pandemic, calling for a fundamental reassessment of how we understand ourselves as a species and how we relate to non-human species. Crucially, he argues that this rethinking must be informed by evolutionary theory and ecological awareness. According to Worster, our biological and cultural drive to control natural phenomena, especially as manifested in large-scale industrial agriculture and livestock production, systematically ignores or wilfully denies the fact that we are embedded within broader evolutionary and ecological processes. By treating living and non-living elements of the planet as mere “natural resources”, we are altering our relationship with the biosphere in radical and destabilising ways.

Naturally, other texts intersect with the present study. In May 2020, I came across a short article, more precisely a correspondence piece, in the prestigious journal *Nature*, titled ‘The Proximal Origin of SARS-CoV-2’.^6^ As the title suggests, the article focuses on the proximate origins of the coronavirus responsible for the COVID-19 pandemic. It does so by comparing SARS-CoV-2 with other phylogenetically related viruses, such as SARS-CoV (linked to the 2002-2003 outbreak), MERS-CoV (2015), and a coronavirus isolated from pangolins, in order to argue that SARS-CoV-2 is unlikely to have originated from a laboratory incident. This article occupies a central position in the still-unresolved debate concerning the origins of SARS-CoV-2.^6^ ^7^ ^8^ ^14^ ^15^ For those engaged in this scientific and social controversy in good political and ethical faith, understanding how the virus emerged and crossed the species barrier is essential for preventing future pandemics. Although the laboratory-leak hypothesis has been significantly weakened, the exact pathway by which the virus reached humans, likely from bats and possibly via intermediate species sold in live animal markets, remains unknown.^9^ ^10^

The problem of the origin of SARS-CoV-2 quickly became entangled in geopolitical disputes, extending beyond scientific and epidemiological inquiry into arenas of strategic communication and influence. Competing narratives circulated not only through scientific channels but also across digital platforms such as Instagram and Twitter, where they were amplified, simplified, and often politicised. While these environments facilitated the spread of misinformation and the reframing of uncertainty as suspicion, they also enabled rapid, open, and sometimes highly productive exchanges among scientists, journalists, and informed publics. As a result, the investigation of origins unfolded within a hybrid space shaped simultaneously by scientific evidence, geopolitical interests, and the ambivalent dynamics of social media circulation, thereby complicating any straightforward attribution of causality.^7^ ^15^

In the next section of this study, I examine Caponi’s experimentalist conception of causality, drawing on a brief yet significant passage in which he addresses a health-related case. Building on this foundation, I propose a manipulationist account of the COVID-19 pandemic, one that highlights our capacity to intervene in the dynamics of such crises. I then turn to Donald Worster’s central claim: that humanity’s compulsion to control natural systems, amplified by massive population growth and the exponential increase in global mobility, has placed us in a precarious and unstable position. This condition, I argue, reflects a paradox of control, in which efforts to master nature generate new vulnerabilities, including the conditions for pandemic emergence. For Worster, any attempt to resolve this dilemma must take seriously the relevance of evolutionary theory and ecology to human life. I conclude by suggesting that a meaningful explanation of the COVID-19 pandemic must not only account for its causes, but also contribute to the prevention of future catastrophes. If, as Caponi suggests, explaining is inseparable from doing, experimenting, and intervening, then causal understanding becomes a tool for action. And if action without understanding risks deepening the crisis, explanation must be oriented towards foresight, responsibility, and care.

From this perspective, the history of human transformations of ecological systems can itself be interpreted as a large-scale field of interventions in evolutionary and ecological processes, even when these interventions occur without the experimental control that characterises scientific practice.

## RESULTS AND DISCUSSION

*Caponi meets Thagard: causal explanation without laws* - *Leyes sin causa y causas sin ley en la explicación biológica* (*Leyes sin causa* hereafter), published in 2014 by the Argentine philosopher of biology Gustavo Caponi, addresses core debates in the philosophy of biology, with a focus on causal explanation in evolutionary and ecological contexts. A prolific scholar currently based at the Federal University of Santa Catarina in Brazil, Caponi has authored more than 150 articles and over a dozen books. In *Leyes sin causa*, he begins by examining the limitations of the nomological-deductive model (NDM) when applied to biological phenomena. According to the NDM, a phenomenon is explained when it can be logically deduced from general laws and initial conditions.^16^ Within this framework, causal explanation consists of the subsumption of events under general laws, allowing for the deduction, prediction, and logical reconstruction of outcomes.

Caponi critically engages with a wide range of philosophical literature, drawing most notably on James Woodward’s interventionist^17^ model and Henrik von Wright’s^18^ analysis of causality. He begins with a seemingly straightforward example from physics, Archimedes’ principle, which describes the relationship between the volume of a submerged object and the density of the fluid it displaces. This example serves as a springboard for a broader reflection on the status of causal laws in the life sciences. An important distinction proposed by Caponi concerns the difference between causal laws and consequential laws. Causal laws specify a direct connection between two events or states of affairs: a particular condition (the cause) leads to another (the effect). They provide explanatory power by identifying the mechanisms that produce outcomes. Consequential laws, by contrast, express regularities or patterns observed in nature without necessarily implying a causal mechanism. They describe what tends to happen, but not why it happens.

This distinction between consequential and causal laws is particularly significant in biology, where many observed patterns, such as adaptations and species distributions, often reflect statistical tendencies rather than strict universal laws. Caponi’s analysis underscores the limitations of importing explanatory models from the physical sciences into evolutionary biology and argues for alternative frameworks of causal reasoning better attuned to the historical and contingent nature of biological systems. Caponi identifies several difficulties faced by philosophers of science, particularly those working in biology, when attempting to determine what qualifies as a causal explanation. Much of the challenge lies in the elusive character of causal laws in evolutionary and ecological contexts, where phenomena are typically transient, historically contingent, and locally specific. These characteristics undermine the applicability of universal, exceptionless regularities as the foundation of scientific explanation.

From the outset, however, Caponi gestures towards a conceptual alternative, one that sidesteps the demand for universality while remaining philosophically coherent. On page 128 of *Leyes sin causa*, he draws on Paul Thagard’s influential book *How Scientists Explain Disease*,^19^ a classic in the philosophy of biology and medicine. Thagard analyses the historical development of causal models for peptic ulcers, tracing the shift from psychosomatic explanations involving stress and excess stomach acid to the discovery that many cases are caused by infection with the bacterium *Helicobacter pylori*. Importantly, Thagard arrives at this explanation not simply by asserting biological mechanisms, but by offering a historically layered and epistemologically informed account of how explanatory models evolve. He uses diagrammatic schemas composed of relational concepts and arrows to show how causal complexity increases over time, especially as non-biological factors are incorporated. For Caponi, the case of gastric ulcers exemplifies how causal explanations in biology can be robust and operationally effective without appealing to universal laws:

Pero, si existe un ejemplo de explicación causal biológica que definitivamente no encaja, ni en la idea de heteronomía explicativa, ni tampoco en el MND, ese ejemplo es el de la explicación de las enfermedades infecciosas. Tal es el caso de la explicación bacteriológica de muchas úlceras gástricas propuesta y aceptada bajo el imperio de la biología Molecular, pero atendendo a coordenadas típicas de la Microbiología Clásica: la Microbiología de Pasteur y de Koch. Sin tener que valer para todas las úlceras, ni siquiera para todas las posibles úlceras infecciosas, esa explicación estableció un invariante, muy estable aunque tal vez de aplicación muy restringida, que permite explicar y tratar un tipo particular de esa patologia.^12^

[But if there is one example of a biological causal explanation that definitely fits neither the idea of explanatory heteronomy nor the MND, it is the case of the explanation of infectious diseases. Such is the case of the bacteriological explanation of many gastric ulcers, proposed and accepted under the dominance of Molecular Biology, yet guided by coordinates typical of Classical Microbiology: the Microbiology of Pasteur and Koch. Without having to account for all ulcers, nor even for all possible infectious ulcers, that explanation established an invariant - highly stable, though perhaps of very limited applicability - that makes it possible to explain and treat a particular type of this pathology.]

In this context, explanatory heteronomy refers to the claim that “organic phenomena must be explained by resorting to laws originating in physics and chemistry”.^12^ Caponi explicitly rejects this view, while acknowledging that “we are all physicalists”^12^ in the sense that “we accept that every causal relationship must be embodied or anchored in a physical substrate or support”.^12^ The concept of an invariant, central to Caponi’s conception of causality, will be addressed shortly. Before doing so, however, it is important to clarify what constitutes a bacteriological explanation for many cases of gastric ulcer:

En la década que va de 1985 a 1995, los médicos australianos Robin Warren y Barry Marshall consiguieron mostrar que *Helicobacter pylori* era el agente responsable de muchas de esas úlceras en virtud de tres conjuntos de datos: [1] evidencia clínica y epidemiológica de la asociación entre dicha bacteria y esa patología; [2] éxito en la producción experimental de la úlcera por inoculación de cepas puras, cultivadas experimentalmente, de la bacteria imputada; y [3] evidencia experimental y clínica creciente de cura de la úlcera por el uso de un antibiótico específico. Es decir: la úlcera se puede producir inoculando *Helicobacter pylori*, y se puede curar eliminando ese agente. Nadie precisa ninguna ley, y menos una ley física o química, para justificar las imputaciones causales que a partir de ahí puedan formularse sobre casos clínicos concretos.^12^

[In the decade between 1985 and 1995, the Australian physicians Robin Warren and Barry Marshall succeeded in demonstrating that *Helicobacter pylori* was the causal agent responsible for many of these ulcers, based on three sets of evidence: [1] clinical and epidemiological data showing the association between this bacterium and the pathology; [2] the successful experimental production of ulcers through the inoculation of pure, laboratory-cultivated strains of the implicated bacterium; and [3] growing clinical and experimental evidence of ulcer cure through the use of a specific antibiotic. In other words: ulcers can be produced by inoculating *Helicobacter pylori* and can be cured by eliminating this agent. No general law - least of all a physical or chemical law - is needed to justify the causal imputations that can be formulated from this point on in relation to concrete clinical cases.]

The bacteriological explanation operates at a level of analysis distinct from the physical and chemical strata. Although materially grounded in these lower levels (“we are all physicalists”), it stands as a legitimate causal explanation without having to invoke them directly. According to Caponi, such an explanation does not require the statement “*H. pylori* causes peptic ulcers” to be supported by a universal, exceptionless law. Instead, it is validated by an invariant proposition linking cause and effect, experimentally tested, for example, when the bacterium is inoculated into experimental animals that subsequently develop the disease, and when antibiotics targeting *H. pylori* are administered to humans, leading to the disappearance of the ulcers. Through this specific intervention, Barry Marshall and Robin Warren discovered a method for experimentally manipulating the disease. “To explain is to discover a way to do: it is re-doing with thought”.^12^ For Caponi, manipulation constitutes “something like the primitive core, the seed from which the idea of causation has developed”.^12^ Causality, then, is not grounded in a universal law but in an invariant that enables consistent and relatively stable relationships between variables:

La idea de control experimental conlleva una noción que es crucial en la concepción experimental de la causación: aludo a eso que Woodward (...) llama ‘invariancia’ (...). Intervención e invariancia son, em efecto, dos nociones estrechamente vinculadas. Los estados de una variable solo pueden ser controlados por intermedio de la manipulación de los estados de otra variable, que sea realmente distinta a la primera, si entre esos estados existe una asociación constante tal que pueda preverse la modificación que ocurrirá en la variable controlada en virtud de nuestro conocimiento de la modificación que introduciremos en lavariable de control.^12^

[The idea of experimental control entails a notion that is crucial to the experimental conception of causation: I refer here to what Woodward (…) calls ‘invariance’ (…). Intervention and invariance are, in fact, two closely related notions. The states of a variable can only be controlled through the manipulation of the states of another variable - one that is genuinely distinct from the first - if there exists a constant association between those states, such that we can predict the modification that will occur in the controlled variable by virtue of our knowledge of the modification we introduce into the control variable.]

If I interpret Caponi’s terminology correctly, to causally explain the pandemic would involve identifying both ontological conditions and counterfactual epistemological claims that imply the possibility of manipulating or experimentally controlling COVID-19, serving as either empirical or mental tests of the phenomenon to be explained. Counterfactuals are “if...then” statements that describe alternative scenarios, ranging from general to highly specific, that did not occur but remain logically coherent within the relevant system of variables. Invariances, whether local or more general, govern the behaviour of variables under intervention. “Invariance under interventions” (“invariancia bajo intervenciones”) is the cornerstone of Caponi’s argument: “To explain something causally always necessarily involves a counterfactual presumption about how things would have been if that which is identified as the cause had not occurred in the way it did”.^12^ How might the COVID-19 pandemic have unfolded had no public health measures been implemented? What consequences would have followed, for instance, if masks had never been used?

There is, for example, substantial evidence for a causal relationship, at the population level, between the variables “mask use” and “number of cases”.^20^ ^21^ ^22^ In most cases, increased mask use leads to a reduction in the number of infections, an example of an invariant relationship, or invariance. This consistency between intervention (mask use) and outcome (fewer cases) allows for the effective manipulation of the epidemiological context. Although controlled experiments involving humans are ethically impermissible and multiple variables influence infection rates, the effectiveness of mask use remains measurable through observational and statistical methods. This kind of idealised case supports the identification of causal agents of the pandemic, such as the virus itself and its transmission routes. In addition to mask use, interventions such as social isolation, quarantine, contact tracing (particularly in the early phases of transmission, as noted by Roger Frutos^14^), testing, and vaccination alter both the viral and the human environment. These actions modulate the selection pressures acting on the pathogen and help to control its transmission dynamics.

It becomes important to recognise that different explanatory traditions within and beyond biology operate with partially divergent causal frameworks. The manipulationist conception of causation emphasised by Caponi highlights the role of interventions and invariant relations between variables as the basis of causal explanation. At the same time, Caponi situates biology among the so-called “special sciences”,^12^ whose explanatory practices do not rely on universal laws but on context-dependent generalisations and heterogeneous causal strategies. In this sense, approaches developed in fields such as Political Ecology, Environmental History, and Public Health can be understood as operating within this broader domain of the special sciences, often emphasising structural socio-environmental determinants, including economic systems, land-use transformations, and patterns of global mobility.^1^ ^23^ ^24^ To what extent do these frameworks align with experimental or interventionist models of causation? As Caponi himself notes, biology frequently accommodates heterogeneous explanatory strategies whose epistemic foundations are not fully commensurable. The dialogue proposed in this article between Caponi and Donald Worster therefore unfolds precisely within this tension, seeking to articulate biological, ecological, and historical dimensions of causation without reducing one explanatory register to another. These partially incommensurable epistemic frameworks are not merely theoretical: they imply divergent preventive strategies, ranging from targeted biological interventions to structural transformations in socio-environmental systems, each grounded in distinct causal assumptions and political commitments.

*Caponi meets Worster: artificial selection and uncontrolled control* - The key point in the dialogue I propose between Caponi and Worster is that natural and social phenomena are, to a significant extent, susceptible to control and manipulation. It is useful to clarify the notion of “uncontrolled control” that frames the dialogue proposed in this section. Uncontrolled control refers to a form of intervention in which the capacity to manipulate biological and ecological systems systematically generates outcomes that exceed predictive and regulatory capacities. In Caponi’s experimentalist account, control refers primarily to the possibility of intervening in a system in order to identify invariant causal relations between variables. Control, in this sense, is epistemic and methodological: it enables scientists to manipulate phenomena and thereby understand their causal structure. Worster, by contrast, uses the language of control in a historical and ecological sense. For him, modern societies have progressively expanded their capacity to reorganise ecosystems through agriculture, industrial production, and the large-scale transformation of landscapes. The notion of “uncontrolled control” emerges precisely at the intersection of these perspectives. Human interventions designed to control biological and ecological processes often generate complex evolutionary and environmental dynamics that escape deliberate regulation. In this sense, the same capacity for intervention that makes causal explanation and technological mastery possible also contributes to the production of new forms of instability, including the ecological conditions that favour the emergence of pandemics.

Caponi shows that evolutionary biology provides a compelling example, widely acknowledged but rarely analysed in this particular framework, in which interventions targeting processes that constitute the so-called invariants of natural selection make it possible to control phenomena of major relevance to public health:

La selección está más a mano de lo que algunos parecen creer. Y el hombre ha operado con ella desde que comenzó a seleccionar las variedades de las especies domésticas. La selección artificial es una presión selective ejercida por nosotros mismos: ella no es un fenómeno análogo a la selección natural, ella es selección natural. Como ha dicho Robert Brandon (...): “la selección artificial es solo un tipo de selección natural”.^12^

[Selection is more readily available than some seem to believe. Humans have operated through it ever since they began selecting varieties among domestic species. Artificial selection is a selective pressure exerted by ourselves: it is not a phenomenon analogous to natural selection; it is natural selection. As Robert Brandon has stated (...): “artificial selection is just one type of natural selection”.]

In other words, artificial selection is a method developed by humans to manipulate animal and plant populations, shaping their traits with far-reaching implications for the exploitation of so-called “natural resources” for human benefit. Agriculture and animal husbandry, each serving distinct purposes, are grounded in this practical knowledge: across generations, certain animal or plant individuals are selectively bred as progenitors of the next generation, thereby intervening in and controlling a natural process structured by invariances.

Artificial selection plays a pivotal ontological and epistemological role in Caponi’s argument. It illustrates how humans have historically controlled the physical and behavioural characteristics of various animal, plant, and even microbial species, often without any awareness of the underlying biological mechanisms, as in the cases of bread, beer, wine, cheese, and fermented foods.^25^ This manipulation of species relies on a causal relationship: by intervening in the controlling variable, namely the selection of desirable traits observed in one generation, the outcome variable consistently responds through the recurrence, and often the enhancement, of those traits in subsequent generations. The invariance expressed under these interventions is the process of selection itself, here, human selection, which must be sustained over time in order to maintain the desired characteristics.

The manipulation of species, now operating on mega-industrial scales, has contributed to the unintended proliferation of species across distinct biological phyla, including arthropods (*e.g.*, insects), fungi, and various microorganisms (including viruses), alongside vertebrates such as rodents and bats. Many of these organisms are classified as threats to agricultural productivity and are therefore targeted for eradication. Caponi identifies the control of so-called “pests” as yet another instance of the manipulation of living organisms driven by economic and demographic imperatives:

El control biológico de plagas y malas hierbas, lo sabemos, es una tecnología humana muy difundida y relativamente avanzada. Yo solo estoy refiriéndome a ella para destacar cómo, por medio de ese control, y en posible conjunción con el control de factores abióticos que también afectan a los seres vivos, es posible manipular y controlar no solo el crecimiento de ciertas poblaciones, sino también controlar, manipular, revertir o acentuar las presiones selectivas que pueden incidir sobre dichas poblaciones. Lo primero es un control puramente ecológico, pero lo segundo ya es un control de la evolución. Un control de los procesos evolutivos que nos habla de la estabilidad bajo intervenciones de la que gozan los invariantes selectivos que la Teoría de la Selección Natural nos lleva a identificar, para, conbase en ellos, explicar la generación de las presiones selectivas que citamos como causas de esos processos.^12^

[The biological control of pests and weeds, as we know, is a widely disseminated and relatively advanced human technology. I refer to it here only to highlight how, through such control - and possibly in conjunction with the control of abiotic factors that also affect living beings - it becomes possible not only to manipulate and regulate the growth of certain populations, but also to control, manipulate, reverse, or intensify the selective pressures that may act upon those populations. The first is a purely ecological form of control, but the second is already a control of evolution itself: a control of evolutionary processes that points to the stability under interventions enjoyed by the selective invariants that the Theory of Natural Selection leads us to identify, and on which we rely to explain the generation of the selective pressures we cite as causes of those processes.]

For approximately 200,000 years, humans lived as hunter-gatherers, depending on hunting, fishing, and the gathering of fruits, nuts, and roots. The emergence of food production marked a fundamental transformation in human history, based on artificial selection and the domestication of plants and animals, processes that reshaped wild species in accordance with human purposes. Long before Darwin and Wallace formulated the theory of Natural Selection, early farmers, breeders, and naturalists applied techniques of selection and crossbreeding to propagate desirable traits across generations. Plant domestication began around 12,000 years ago, during the Neolithic Revolution, when human societies shifted from foraging to farming. Early agriculturalists systematically selected seeds from high-yielding, resilient, and easily harvested plants, initiating independent centres of domestication in regions such as the Fertile Crescent, the Yangtze River Valley, and the Americas. This transition, facilitated by the climatic stability that followed the last Ice Age, led to the cultivation of staple crops such as wheat, barley, rice, and maize, alongside the domestication of animals including sheep, goats, pigs, and cattle. The development of agriculture underpinned the rise of villages, cities, and complex civilisations, supported by technological innovations such as irrigation systems, ploughs, and crop rotation.^26^ ^27^

Agricultural practices have evolved over centuries. In medieval Europe, the introduction of the three-field system improved soil fertility and crop rotation. During the Renaissance, the arrival of New World crops such as potatoes and maize expanded food availability and nutritional diversity. The Industrial Revolution marked a turning point, as mechanisation, tractors, ploughs, and chemical fertilisers greatly increased agricultural productivity. All of these elements are interconnected through long-distance transportation infrastructures that rely on the combustion of fossil fuels, an arrangement that extends human control across time and space while simultaneously deepening dependence on non-renewable resources and accelerating ecological instability. In the 20th century, the Green Revolution brought further intensification through high-yielding crop varieties, synthetic fertilisers, and pesticides. While these innovations reduced hunger in many regions, they also accelerated soil degradation, resource depletion, and biodiversity loss. These transformations underscore the extent to which human activity has reshaped ecological systems and altered selective pressures across ecosystems, generating both gains in productivity and long-term risks to environmental sustainability.

The mid-20th century also marks what historians and scientists call the Great Acceleration, a phase of intensified industrial activity and resource extraction with profound and measurable impacts on climate and ecosystems. This process was driven primarily by fossil fuels, as well as by a historically specific configuration in which scientific knowledge and technological innovation became deeply intertwined with capitalist modes of production, particularly in their monopolistic and fossil-fuel-dependent forms. In this context, science and technology function as engines of planetary transformation, enabling the expansion of industrial agriculture, global supply chains, and extractive economies that have accelerated environmental change. These developments have been widely interpreted as marking the emergence of the Anthropocene, defined by human-induced transformations of planetary systems and the rise of environmental dynamics with increasingly unpredictable consequences.^28^ ^29^ ^30^ In this sense, “uncontrolled control” captures the manner in which interventions aimed at stabilising biological processes systematically generate new layers of ecological instability across scales.

*Donald Worster meets the Anthropocene* - Donald Worster is widely recognised as one of the founding figures of Environmental History. His scholarship investigates the ways in which human societies have interacted with the natural world over time, analysing both ecological transformations and their cultural, economic, and social ramifications. Among his most influential works is *Shrinking the Earth: The Rise and Decline of American Abundance*,^24^ which tests the theory, in the context of United States history, that there are no limits to what can be extracted from and expected of the natural world. In *Nature’s Economy: A History of Ecological Ideas*,^31^ Worster traces the development of ecological thought from the Enlightenment to the contemporary era, offering a nuanced account of the interplay between ecological science and social values. Worster’s approach is distinctly critical: he contends that the unchecked exploitation of natural resources has produced severe and often irreversible consequences for both ecosystems and human communities. His work challenges linear narratives of progress and modernisation, advocating instead for a reconfiguration of human-nature relations grounded in ecological sustainability.

In April 2020, Donald Worster launched the virtual exhibition *Another Silent Spring* on the “Environment & Society Portal”.^13^ The exhibition presents a curated collection of photographs and visual materials related to COVID-19, accompanied by a sharply articulated essay by the environmental historian. At that point, more than 150,000 people had died across multiple countries on all five continents. City streets around the world stood empty, silenced by government interventions intended to halt the unchecked spread of a novel virus with no prior circulation in human populations, what Alfred W. Crosby might have described as a “virgin soil pandemic”.^32^ Worster draws a poignant parallel between the spectral stillness of cities in 2020 and the ecological silence evoked by Rachel Carson’s *Silent Spring*,^33^ in which the widespread use of pesticides threatened to erase the natural soundscape. In both cases, the silence signifies not peace but disruption, an absence shaped by human attempts to intervene in and control natural processes. *Silent Spring* opens with a parable describing a rural American landscape rendered biologically barren, its domestic and wild species eradicated by chemical agents we now classify as herbicides and insecticides. Carson invites readers to imagine the haunting silence that envelops this fictionalised setting and, while acknowledging its imagined nature, draws attention to the widespread and very real application of chemical substances across American farmland. The book ignited a national debate over the risks posed by synthetic chemicals, prompting critical reassessment of their use and leading to the implementation of more rigorous environmental regulations. *Silent Spring* played a pivotal role in the eventual ban on DDT in the United States in 1972 and in the creation of regulatory institutions such as the Environmental Protection Agency. Carson emphasised the interdependence of living systems, demonstrating how human interventions can destabilise food chains and disrupt ecological cycles. Often regarded as a founder of the modern environmental movement, she catalysed widespread environmental awareness and activism, underscoring the inseparability of environmental and human health.

In 1962, when *Silent Spring* was published, the global population was approximately 3 billion. Yet concerns over large-scale food production and distribution were already acute. The expansion of monoculture agriculture and industrial animal farming had created novel ecological niches occupied by various insect species, other arthropods, small vertebrates, and, less visibly, microorganisms. Rachel Carson does not oppose the underlying goal of managing pests in agriculture and livestock production. Rather, she exposes the widespread biological destruction caused by chemical control methods, including the poisoning and death of countless non-target species and human beings through both direct exposure and indirect contamination. In place of such reactive measures, Carson advocates biologically informed strategies, including insect sterilisation and the use of natural predators, methods aimed at restoring ecological balance rather than disrupting it.

The global human population has now surpassed eight billion, with projections indicating a rise to eleven billion by the end of the century.^34^ Reflecting on the silence imposed by social distancing and quarantine measures during the early stages of the COVID-19 pandemic, Donald Worster underscores the profound food security challenges of our time, a crisis unfolding within a world marked by geopolitical instability and contested borders, and intensified by climatic upheavals. He identifies a critical trade-off between large-scale food production and the emergence of infectious diseases. The ecological simplifications inherent in what he refers to as “Agro”, particularly in the livestock sector, create conditions conducive to the spillover of pathogens from both wild and domesticated species into human populations.^23^ ^31^ ^34^

The ways in which humans manage plants, animals, and soil, transforming vast landscapes into monocultures and pastureland, contribute directly to climate change and introduce new selective pressures on human populations, nonhuman animals, and the vast microbial ecologies within and around them. These shifts have intensified since the mid-twentieth century, coinciding with the Green Revolution and the Great Acceleration.^29^ ^35^ This acceleration is characterised by rapid economic, technological, and demographic expansion, tracked through a range of socioeconomic indicators (such as industrialisation, energy use, urbanisation, and global trade) and environmental indicators (including greenhouse gas emissions, deforestation, biodiversity loss, ocean acidification, and pollution). These interlinked transformations are now widely understood to have disrupted the Earth’s climatic regime, inaugurating a new geological epoch: the Anthropocene.^29^ ^35^ ^36^

For Donald Worster, much of this transformation is rooted in contemporary food systems, particularly in the ways they organise production, distribution, and consumption on a global scale. Central to this transformation is the widespread reliance on fossil fuels, arguably one of the most powerful means ever devised by human ingenuity to harness energy and the generation of material wealth. The discovery, exploitation, and combustion of coal, oil, and gas enabled an unprecedented expansion of industrial agriculture, mechanised transport, refrigeration, and long-distance supply chains, all of which are deeply embedded in modern food systems.^23^ ^28^ ^29^ Yet this same system, while delivering abundance for some, has also deepened global inequalities and contributed significantly to ecological degradation. Fossil fuels epitomise the paradox of control: a form of mastery over nature that accelerates both economic accumulation and planetary instability:

We pursue agricultural development (most recently the Green Revolution); every continent except Antarctica is now under its reign, and food abundance flows endlessly to the cities. So far the strategy has worked well for our kind; we now number nearly eight billion, most of us well fed, hale and hearty. More than half of us can now live in towns, cities, and megalopolises because our efficient world food system supports us.^13^

However, the entire industrial apparatus underpinning the production and distribution of living organisms, both plant- and animal-based, has introduced conditions that would have been unimaginable in earlier periods. What once appeared to be an unequivocal achievement, namely the control of the reproduction of domesticated species in order to enhance nutrition and abundance, now reveals its hidden costs, some of which are measured in human lives:

Agriculture, though born of necessity, is the essential foundation of civilization, including written languages, the state, and the city. Modern industrialization has built itself on the back of the farmer and cannot live without his food surpluses. As agricultural settlements began to trade with one another, they unwittingly spread viral mutations and cause other problems far from home. Thousands of years later, those busy with local concerns may not know the distant consequences of their actions.^13^

Donald Worster identifies industrial agriculture as an immediate driver of increased human contact with animals carrying viruses and other parasites that would otherwise remain largely confined to their wild reservoirs. Industrial agriculture can therefore be understood as a paradigmatic case of uncontrolled control: a system built upon the deliberate manipulation of biological processes whose large-scale ecological consequences exceed the intentions and predictive capacities of those who manage it. At the same time, he identifies the very emergence and development of agriculture as a more distant structural cause, an expression of the enduring human impulse to control and manipulate natural processes. He articulates this point in two ways, the first through an analogy with Carson’s *Silent Spring*:

By driving agricultural production to new heights, consumers threatened their own health as well as the health of the planet. (Carson linked rising cancer rates and other modern diseases to the new pesticides.) The ultimate cause, therefore, was not simply a concentration of economic and technological power; it was a deeper and broader cultural drive to conquer nature. While it was true that some powerful agribusiness companies had led the creation of new agricultural chemicals, showing “no humility before the vast forces with which they tamper,” ordinary people bore some responsibility too. Millions of people had gladly bought and used those chemicals or otherwise supported their use. The domination of nature was what they commonly sought, and chaos was what they got.^13^

In the interpretation advanced here, this “deeper and broader cultural drive to conquer nature” is closely linked to Caponi’s conception of causality as grounded in control, experimentation, intervention, and manipulation. From this perspective, the impulse to dominate natural systems may reflect adaptive cognitive and cultural tendencies shaped over evolutionary time, particularly when considering how cultural behaviours may emerge and stabilise in response to long-term environmental pressures. As is widely recognised, Natural Selection favours traits and behaviours that enhance survival and reproductive success. In the case of humans, the capacity to manipulate the environment through tool use, agriculture, fire, shelter construction, and other techniques represents a significant adaptive advantage. Cultures that promote the mastery of natural processes likely enabled human groups to survive in diverse and often inhospitable environments, facilitating the exploitation of resources on unprecedented scales. Over time, such practices became deeply embedded within cultural systems, evolving from immediate survival strategies into a broader cultural imperative, one expressed through the pursuit of progress, technological development, and dominion over nature. To the extent that these behaviours improved the fitness of populations, they may have been reinforced by natural selection, giving rise to culturally transmitted dispositions that continue to shape human relationships with the biosphere.

The modern drive to control natural processes cannot be understood solely as a cultural orientation or as an adaptive strategy for survival in hostile environments. It has been historically reinforced by the alliance between scientific knowledge, technological innovation, and capitalist forms of production, particularly under fossil-fuel-dependent and large-scale industrial regimes.^28^ ^37^ These socio-technical arrangements have greatly expanded human capacities to reorganise ecosystems and transform evolutionary and ecological conditions, thereby reshaping the selective pressures acting upon other organisms, including pathogens.

As observed in Rachel Carson’s toxic spring and Donald Worster’s viral spring, the pursuit of control over “resources” has spiralled out of control, with catastrophic consequences. In this context, Worster argues that if we continue to disregard ecology and the theory of Natural Selection, we are doomed to repeat the mistakes that led to COVID-19:

Yet most of us remain uneducated in the science needed to understand epidemic history: the Darwinian revolution has given us astounding new knowledge that can explain more profoundly than ever our history on earth. Yet even now, ignorance, or cultural resistance, characterizes many citizens, including some university professors, corporate executives, and even experts in public health. They simply haven’t learned to think about disease in Darwinian terms, and thus they are doomed to repeat the ultimate causes of all the dying that keeps happening.^13^

According to Donald Worster, the significance of disciplines such as Evolutionary Biology and Ecology lies, in part, in their emphasis on the interconnectedness of human life with the broader natural world. These fields challenge anthropocentric assumptions that soil, animals (including humans), and plants exist solely as resources to be exploited under capitalist and neoliberal logics.^28^ ^35^ ^37^ Once again, the impulse to control and manipulate natural processes, perhaps not an exclusively human trait, may be understood as an evolutionarily adaptive feature of the mind. Yet for Worster, this impulse has become maladaptive: by intensifying selective pressures acting upon pathogens with pandemic potential, it contributes to conditions that favour spillover events and facilitate viral transmission. Random mutations enabling efficient human-to-human transmission now encounter highly favourable conditions in a densely populated and hyperconnected world, one characterised by frenetic human movement through aeroplanes, airports, stadiums, transport hubs, and commercial and domestic spaces across local, regional, national, and international scales. In effect, humanity has unintentionally designed an uncontrolled global experiment through its management of plants and animals. In seeking to dominate natural processes for human benefit, humans have created ecological conditions that support the emergence and proliferation of novel pathogens with the potential to trigger future pandemics.

In his collection of essays *Big Farms Make Big Flu: Dispatches on Infectious Disease, Agribusiness, and the Nature of Science*,^23^ the American evolutionary epidemiologist Rob Wallace argues that the global system of agricultural production, driven by profit maximisation, constitutes a central force behind the emergence and spread of novel infectious diseases. According to Wallace, industrial farming practices create conditions conducive to the accelerated evolution of more dangerous viral strains. High animal density, genetic uniformity, prophylactic medication, and chronically stressful environments foster the emergence of pathogens that are both more virulent and more resistant to treatment, thereby increasing the likelihood of large-scale outbreaks in human populations. In *Big Farms Make Big Flu*, Wallace presents H5N1 as a paradigmatic case illustrating the accelerated evolution of virulent strains in intensive monocultural environments. He traces the global dissemination of the virus through industrial farming practices and underscores the urgent need for public policy and alternative agroecological models capable of disrupting this pandemic-selection cycle. Unsurprisingly, one of today’s most pressing epidemiological concerns is the potential emergence of an H5N1 variant capable of sustained human-to-human transmission, especially given the virus’s ongoing spread among aquatic birds, marine mammals, and terrestrial wildlife.^38^

Rob Wallace proposes that smaller-scale, decentralised, and ecologically grounded models of food production, anchored in biodiversity and local autonomy, could significantly reduce the risk of future pandemics. The fact that industrial farms, particularly poultry operations, function as incubators for respiratory pathogens illustrates how the manipulation of animal reproduction under conditions of extreme confinement, with crowded animals breathing the same air, amounts to an uncontrolled evolutionary experiment in virulence. Throughout his work, Wallace systematically identifies the corporate structures underpinning the global meat industry, exposing how they exploit traditional farmers while operating with substantial support from public subsidies. While it is well established that SARS-CoV-2 did not originate directly from an industrial farm, its emergence is, as Donald Worster argues, intrinsically linked to the ways in which humanity exploits so-called “natural resources”.

*In conclusion* - How did bat-borne viruses reach human hosts? And why did this one, in particular, achieve pandemic status? The precise transmission pathway through which SARS-CoV-2 entered human populations remains unknown. Yet even without identifying its exact origin, we were able to exert a degree of control over the virus’s evolution and impact. Through quarantine, isolation, masking, vaccination, and other public health interventions, societies managed to mitigate the severity of the pandemic and reshape its trajectory. In this sense, the pandemic was causally explained in a philosophically coherent manner: through intervention in a complex biological and social phenomenon, humans acted upon future outcomes, increasing the likelihood of improved public health conditions. At the same time, these interventions may have inadvertently selected for viral variants better adapted to the conditions imposed upon them, thereby altering the virus’s evolutionary trajectory. Despite these efforts, COVID-19 now appears likely to remain with us for the foreseeable future, woven into the biological and social fabric of human life.

In *New pandemics, old politics*,^1^ the British sociologist Alex de Waal explores the militarisation of public health through the recurring metaphor of war against microorganisms. Drawing on historical episodes in which infectious diseases have alarmed populations worldwide, from tuberculosis and the Spanish flu to human immunodeficiency virus/Acquired Immunodeficiency Syndrome (HIV/AIDS) and COVID-19, he shows how responses to large-scale outbreaks have often been framed within a bellicose logic of conquering nature. According to de Waal, the metaphysical frameworks through which we interpret etiological agents shape our interactions with them.^21^ The view of bacteria and viruses as hostile enemies to be eradicated neglects the fact that these organisms, even when pathogenic, are simply participating in the Darwinian struggle to maximise their own replication. In our zeal to eliminate them, we may unwittingly promote their evolution, an effect evident in the development of antibiotic resistance among bacteria and pesticide resistance among agricultural pests.

This adversarial stance obscures the ecological reality: bacteria, viruses, insects, arthropods, and countless other organisms form part of the intricate web of life to which humans also belong. By reducing them to economic or biomedical threats, we disregard their ecological roles and the broader systems upon which our own health depends. Drawing on the work of physician and anthropologist Rudolf Virchow, Alex de Waal highlights what he describes as the “indissoluble connection between medical science, economic interest, and political ideology”. As Virchow famously stated, “medicine is a social science, and politics is nothing more than medicine on a large scale”.^1^ For de Waal, both medical science and political institutions must be critically informed by evolutionary theory and ecological understanding.

In a 2022 study, Bernstein and colleagues argued that the number of lives lost and the economic costs associated with viral zoonotic pandemics have increased markedly over the past century. In response, public policies in various countries have begun to prioritise strategies aimed at detecting and containing emerging zoonotic threats. However, the authors contend that such interventions are typically implemented only after outbreaks are already underway, a reactive approach they fundamentally reject. Human populations maintain extensive contact with wildlife species known to harbour an astronomical diversity of microorganisms, including viruses to which human populations remain immunologically naïve, in the “virgin soil” sense.^32^ Bernstein and colleagues examine the impacts of emerging viral zoonoses and, through a counterfactual lens, propose three actionable strategies aimed at manipulating key variables in order to prevent future pandemics: enhanced surveillance of pathogen spillover, including global databases for viral genomics and serology; improved regulation of wildlife trade, particularly illicit networks; and substantial reductions in deforestation. They conclude that the cost of implementing these primary prevention measures would amount to less than one-twentieth of the annual economic losses attributed to emerging viral pandemics.^39^ Yet, as with the agroecological reforms advocated by Rob Wallace, pandemic prevention remains a politically fraught endeavour. It not only challenges entrenched economic interests but also suffers from a fundamental epistemological dilemma: how can policymakers convincingly demonstrate that a catastrophe has been averted? How can one prove that a pandemic would have occurred had wildlife trade not been restricted, deforestation not reduced, or industrial agriculture not restructured? This dilemma points to a broader existential challenge, one that demands resolution or threatens to overwhelm us. The commodification of animals, plants, and other so-called “natural resources”, privatising profits while socialising ecological and epidemiological risks,^23^ has emerged as a profound threat to the health and well-being of both human and non-human life.

The connection between Caponi’s and Donald Worster’s perspectives in the context of COVID-19 lies in the tension between control as a means of containment and control as a root cause of crisis. Caponi’s framework helps conceptualise experimental control as a rational and operational strategy for mitigating the effects of the pandemic, demonstrating how human intervention can alter the course of viral evolution. Worster, by contrast, underscores how unrestrained forms of control, manifested in industrial food systems and the intensive exploitation of “natural resources”, played a key role in precipitating the emergence of SARS-CoV-2. The pandemic thus crystallises a paradox of control: while interventions grounded in causal understanding and experimental manipulation (such as vaccines and public health measures) proved essential for mitigating the crisis, the ecological and evolutionary destabilisation produced by global systems of biotic and abiotic extraction also contributed to its emergence. This duality underscores the urgency of rethinking how control is exercised and redesigning it in ways that respect ecological limits and evolutionary dynamics in order to reduce the likelihood of future pandemics.

## Data Availability

No new data was generated or analysed in support of this research.
