# Supplementary material for: The causes of COVID-19: an uncontrolled experiment in evolution, causation, and public health
Source: Mem Inst Oswaldo Cruz. 2026 Oct 26;121(Suppl 1):e250285. doi: 10.1590/0074-02760250285 (PMC13722147; doi:10.1590/0074-02760250285)
Supplement: Peer review history

# Reviewer report: FIRST REVIEW ROUND - REVIEWERS’ COMMENTS

## REVIEWER #1

The manuscript offers an original and intellectually ambitious contribution to ongoing debates about the causes underlying the emergence of Covid-19 and its subsequent pandemic trajectory. By placing a careful reading of contemporary philosophy of biology in dialogue with reflections drawn from environmental history, the article succeeds in framing the pandemic not merely as a biomedical event, but as a problem situated at the intersection of ecological, social, and epistemological processes. This interdisciplinary move is both timely and highly relevant in light of the global health crisis that began in 2019.

One of the article’s main strengths lies in its use of the distinction between proximate and remote causes. This analytical move allows the author to approach the problem of causation in a broader and less dogmatic manner, moving beyond narrowly framed explanations. By articulating debates from biology and epidemiology with perspectives that foreground socioeconomic, political, and cultural dynamics—deeply entangled with the ecology of disease—the manuscript highlights the limits of monocausal or strictly reductionist accounts. The engagement with Gustavo Caponi’s work is particularly effective in demonstrating the epistemic plurality that characterizes the biological sciences themselves, underscoring that no single causal framework can claim universal or exclusive validity.

The manuscript is grounded in a well-chosen and up-to-date body of specialized literature drawn from multiple fields, reflecting both breadth and scholarly rigor. It is especially appropriate that a journal devoted to biomedical research should host an article that seeks to complexify prevailing understandings of disease causation through philosophical, historical, and political-ecological perspectives. In doing so, the article adds an important layer of complexity to contemporary efforts to explain disease emergence—complexity that has become increasingly evident with the rise of pathologies associated with cumulative toxic exposures and other conditions in which causal chains are diffuse, layered, and difficult to disentangle.

For these reasons, I consider the article suitable for publication pending minor revisions, which would further enhance its analytical clarity and internal coherence. The suggestions below are offered in a constructive spirit and are intended to strengthen an already compelling manuscript.

First, I would recommend that the article state more explicitly, from the outset, its central argument and the rationale for bringing together the works of Gustavo Caponi and Donald Worster. As the analysis progresses, it becomes clear that this juxtaposition enables a more comprehensive understanding of the emergence of epidemics and pandemics by articulating biological explanations across different scales—from viral mechanisms to broader ecological and evolutionary processes—while also incorporating political-economic dimensions. This objective is articulated particularly clearly in the conclusion, notably in the statement that a meaningful explanation of the Covid-19 pandemic must not only account for its causes but also contribute to preventing future catastrophes. Bringing this guiding aim forward into the introduction would help frame the analysis more clearly for the reader.

Second, the manuscript would benefit from a slightly more explicit discussion of what might be termed “epistemic incommensurabilities” between different ways of establishing causal relations within biology itself—as highlighted by Caponi—as well as between biological explanations and those developed in the social sciences and humanities. Approaches associated with political ecology, collective or public health, and related traditions often operate with explanatory models centered on socio-environmental determinants that do not easily align with dominant biological frameworks. The article already gestures convincingly toward these tensions; making this point more explicit would further clarify its contribution to debates about prevention-oriented understandings of disease emergence.

Third, it would be useful to further develop, within the main body of the text, the concept of “uncontrolled control” introduced in the subtitle of the relevant section. Given its centrality to the argument, the notion of control—and the divergences, convergences, and frictions between Caponi’s and Worster’s respective understandings of it—would benefit from a more sustained analytical treatment rather than remaining primarily a thematic marker.

I also offer a few more specific suggestions. On page 20, it would be important to explicitly identify fossil fuels as the primary drivers of climate change. While the category of “ecological degradation” does encompass climate change, the magnitude and centrality of climate change—arguably the most dramatic outcome of fossil-fuel-based industrialization—warrant explicit mention.

Relatedly, in the subsequent discussion of “cultural behaviors that emerge and stabilize in response to long-term environmental pressures,” I suggest incorporating a more explicit consideration of science and technology, particularly in their symbiotic relationship with capitalism, especially in its monopolistic and fossil-fuel-dependent forms. An entangled analysis of biological and sociocultural dimensions would be strengthened by acknowledging how technologies produced through the alliance of science and capitalism have amplified human capacity to transform ecologies and impose selective pressures on other organisms. In this sense, the drive toward control and mastery of natural processes has involved not only cultural systems, but also economic, political, and metabolic regimes, extending well beyond the imperative of mere survival in hostile environments.

To further strengthen the link between Covid-19 and industrial agriculture, it might also be helpful to briefly outline, earlier in the article, the main epidemiological hypotheses and forms of evidence that support the pathway from bats to human hosts. At present, this discussion appears primarily in the conclusion; a concise reference in the introduction would help readers better grasp the empirical foundations upon which the broader analytical claims rest.

Finally, while the political dimension of the argument emerges most clearly in the conclusion—particularly in relation to preparedness and the prevention of future epidemics linked to viral spillover—it could be productively reintroduced at other points in the manuscript, especially in the introduction and in the section devoted to Donald Worster’s work. Doing so would further enhance the article’s coherence and sharpen its critical edge.

In this concluding discussion, the manuscript raises important questions about how broader, multicausal understandings of disease emergence might be translated into public health strategies at local, national, and especially global scales. The engagement with the policy prescriptions proposed by Bernstein and collaborators is particularly effective in highlighting the limitations of dominant global health paradigms grounded in restrictive models of causality. As the article rightly suggests, such paradigms may have contributed less to prevention than to the repeated emergence of new viral pathogens.

In this light, the epistemological dilemma identified by Bernstein et al.—namely, how to demonstrate that a catastrophe has been averted—can itself be read as a symptom of deeper philosophical assumptions about causality and evidence. A genuinely multicausal approach does not require “proof” in the strict sense problematized in the opening section of the article. Rather, it calls for a more profound revision of the paradigms that currently shape public health at local, national, and above all global levels—a point repeatedly emphasized by critical voices during the Covid-19 pandemic.

Overall, the comments above concern relatively focused clarifications and modest adjustments to arguments already present in the text. Addressing them would, in my view, further extend the analytical reach and critical force of an article that already makes a significant and timely contribution to the field.

## AUTHORS’ RESPONSE TO THE REVIEWERS

Dear Editor,

I would like to thank you and the anonymous reviewer for the careful and generous evaluation of my manuscript, “The causes of covid-19: a lawless, uncontrolled experiment.” I am especially grateful for the constructive nature of the comments, which have significantly improved the clarity, coherence, and analytical strength of the article.

Below, I detail how each of the reviewer’s suggestions has been addressed in the revised version of the manuscript. The numbering below has been adopted to facilitate the identification of the solutions implemented in response to the reviewers’ suggestions, all of which have been addressed in this revised version of the manuscript.

1. Clarification of the central argument in the introduction

Following the reviewer’s recommendation, I have reformulated the opening section of the manuscript in order to state more explicitly, from the outset, the central argument and the rationale for bringing into dialogue the works of Gustavo Caponi and Donald Worster. The introduction now presents a concise synthesis of the article’s guiding claim: that explaining the covid-19 pandemic requires integrating biological, ecological, and historical dimensions of causation, and that such explanation is inseparable from the development of preventive strategies.

2. Explicit discussion of epistemic incommensurabilities

In response to the second suggestion, I have introduced a more explicit discussion of what the reviewer aptly termed “epistemic incommensurabilities.” This discussion appears both in the introduction and in section 2.1. The revised text now clearly distinguishes between manipulationist causal frameworks in biology and those employed in fields such as political ecology, environmental history, and public health. It emphasizes that these frameworks often operate with partially divergent epistemic foundations and that the dialogue proposed in the article unfolds precisely within this tension.

3. Development of the concept of “uncontrolled control”

The concept of “uncontrolled control,” central to the article’s argument, has been substantially expanded in section 2.2. I now provide a more systematic analytical definition, clarifying how the notion emerges from the intersection between Caponi’s epistemic conception of control and Worster’s historical-ecological understanding of human interventions in nature. Additional passages have been included to show how this concept operates in the context of industrial agriculture and pandemic emergence.

4. Explicit identification of fossil fuels as drivers of climate change As suggested, I have revised the discussion of ecological degradation to explicitly identify fossil fuels as primary drivers of climate change. This clarification has been incorporated into section 2.2, where the relationship between industrialization, energy regimes, and ecological instability is now more clearly articulated.

5. Integration of science, technology, and capitalism

In response to the reviewer’s suggestion, I have expanded the analysis of cultural and ecological transformations to include a more explicit consideration of the symbiotic relationship between science, technology, and capitalism. The revised text now highlights how this alliance has amplified human capacity to transform ecosystems and impose selective pressures, thereby reinforcing the broader argument concerning the historical expansion of control over natural processes.

6. Earlier presentation of epidemiological hypotheses

To strengthen the empirical grounding of the argument, I have added, in the introduction, a concise overview of the main epidemiological hypotheses concerning the emergence of SARS-CoV-2, as well as the types of evidence supporting them (phylogenetics, comparative genomics, ecological studies, and epidemiological investigations). This addition responds directly to the reviewer’s suggestion and improves the accessibility of the argument for readers.

7. Reinforcement of the political dimension

Finally, I have incorporated the political dimension of the argument more consistently throughout the manuscript, particularly in the introduction and in sections 2.1 and 2.2. The revised version now more clearly emphasizes how different causal frameworks entail distinct political and ethical commitments, especially in relation to prevention strategies and the governance of human–environment relations.

All changes are indicated and annotated in the revised version. I believe that these revisions have substantially strengthened the manuscript and clarified its contribution to ongoing debates on causation, pandemic emergence, and prevention. I am deeply grateful to the reviewer for the insightful and precise suggestions, which have played a crucial role in improving the work.

Thank you again for your consideration.

Sincerely,

Ricardo Waizbort

# Reviewer report: REVIEWERS’ COMMENTS

## REVIEWER #1

I consider that the revised version of the manuscript has gained greater analytical clarity, particularly through a more explicit articulation of its central argument and the purpose of the comparative analysis between the two authors and fields of knowledge.

The author has engaged seriously and constructively with the previous round of comments, leading to a substantially improved manuscript. As a result, the article’s contributions to the different fields with which it engages are now more clearly defined and more effectively integrated. In addition, the revisions have helped to refine key formulations concerning the complex interrelations between biological, socio-economic, and cultural processes.

I would like to reiterate that the article represents a highly relevant contribution, not only to ongoing discussions surrounding the COVID-19 pandemic, but also to broader efforts to develop more integrative approaches to understanding the multiple factors that operate in synergy in the emergence, persistence, and control of diseases. Its interdisciplinary perspective, grounded in a sustained, critical, and well-informed dialogue across diverse fields, significantly enriches current debates on health crises in connection with the wider ecological emergency.

## DECISION

DECISION: ACCEPT SUBMISSION | RECEIVED 14 OCTOBER 2025 | ACCEPTED 05 MAY 2026
